# Determinants of vertical transmission of HIV infection in Kilifi County, Kenya: a case-control study

**DOI:** 10.11604/pamj.2026.53.97.49652

**Published:** 2026-02-24

**Authors:** Rukiya Mohammed Koriow, Julius Otieno Oyugi, Christine Muasya, Rodgers Onsomu Moindi

**Affiliations:** 1 University of Nairobi Institute of Tropical and Infectious Diseases (UNITID), Nairobi, Kenya,; 2 Department of Medical Microbiology, University of Nairobi (UON), Nairobi, Kenya,; 3 Department of Clinical Medicine and Therapeutics, University of Nairobi (UON), Nairobi, Kenya,; 4 Department of Health, Research Unit, County Government of Kilifi, Kilifi, Kenya

**Keywords:** Kilifi County, HIV-positive mothers, prevention of mother-to-child transmission, antenatal care

## Abstract

**Introduction:**

mother-to-child transmission of Human Immunodeficiency Virus (HIV) remains a significant contributor to pediatric HIV infections in Kenya despite expanded prevention services. Persistent gaps along the prevention of mother-to-child transmission (PMTCT) cascade continue to undermine elimination efforts.

**Methods:**

we conducted a retrospective, unmatched case-control study using routinely collected PMTCT data from selected health facilities in Kilifi County, Kenya. The cases were HIV-positive mothers whose HIV-exposed infants tested HIV-positive by deoxyribonucleic acid polymerase chain reaction (DNA PCR), while controls were HIV-positive mothers whose infants tested HIV-negative during the same period. Maternal and infant PMTCT cascade indicators were assessed. Associations between PMTCT service uptake and infant HIV status were examined using chi-square tests and multivariable logistic regression.

**Results:**

a total of 161 mother-infant pairs were included (59 cases and 101 controls). Failure to attend antenatal care, lack of maternal antiretroviral therapy (ART) during pregnancy, non-disclosure of HIV status, and home delivery were significantly associated with infant HIV infection. Maternal ART uptake during pregnancy was the strongest protective factor against vertical transmission (adjusted odds ratio 30.23; 95% CI: 7.56-120.96).

**Conclusion:**

missed opportunities along the PMTCT cascade, particularly poor antenatal care (ANC) attendance and delayed or absent maternal ART, remain key drivers of residual mother-to-child HIV transmission in Kilifi County. Strengthening retention, timely ART initiation, and continuity of care across the PMTCT cascade is critical to achieving elimination of vertical HIV transmission.

## Introduction

Human Immunodeficiency Virus (HIV) remains a major global public health challenge, with substantial morbidity and mortality across all age groups. Despite significant progress in HIV prevention and treatment, vertical transmission of HIV continues to account for the majority of new HIV infections among children. Between 2000 and 2024, programs to prevent mother-to-child transmission (PMTCT) are estimated to have averted nearly 4.4 million new HIV infections among children globally [1].

Human immunodeficiency virus transmission from an infected mother to her child may occur during pregnancy, childbirth, or breastfeeding [2]. In the absence of effective interventions, the risk of mother-to-child transmission (MTCT) ranges between 15% and 45%. However, with timely HIV diagnosis, lifelong antiretroviral therapy (ART) for pregnant and breastfeeding women, appropriate infant prophylaxis, and sustained retention in care, MTCT can be reduced to below 5%, the threshold defined for elimination [3]. Sub-Saharan Africa continues to bear a disproportionate burden of pediatric HIV infections due to high HIV prevalence among women of reproductive age and systemic challenges affecting access to and continuity of maternal and child health services [4]. In response, global and regional strategies emphasize a comprehensive PMTCT cascade that includes early HIV testing, prompt ART initiation, viral suppression, safe delivery practices, infant prophylaxis, early infant diagnosis, and sustained follow-up throughout breastfeeding.

**Human immunodeficiency virus and PMTCT in Kenya:** Kenya remains among the countries most affected by the HIV epidemic in sub-Saharan Africa. According to the 2024-2025 national HIV estimates, approximately 1.3 million people are living with HIV, with a national prevalence of about 3.0%. An estimated 62,800 children aged 0-14 years are living with HIV, underscoring the continued contribution of vertical transmission to the pediatric HIV burden [5].

Kenya has made notable progress in scaling up PMTCT services, including universal antenatal HIV testing and the adoption of lifelong ART for all pregnant and breastfeeding women living with HIV. National PMTCT coverage has reached approximately 90%, reflecting substantial improvements in access to services. However, Kenya´s MTCT rate declined only modestly from 10.8% in 2019 to 9.3% in 2024, remaining well above the global elimination target of less than 5% [5]. This trend reflects persistent gaps along the PMTCT cascade, including missed antenatal care (ANC) visits, suboptimal ART adherence and retention, and delays in early infant diagnosis and treatment.

Although approximately 83% of pregnant women in Kenya attend at least one ANC visit, nearly 38% do not complete the recommended four visits, disrupting continuity of care. Furthermore, more than two-thirds of infant HIV infections have been associated with maternal ART non-use or disengagement from care, highlighting the urgent need to strengthen follow-up, adherence support, and service integration to accelerate progress toward elimination of MTCT (eMTCT). To address these challenges, Kenya adopted the national Framework for Elimination of Mother-to-Child Transmission, aligned with global elimination and triple-elimination goals. The framework emphasizes achieving at least 95% performance across key PMTCT cascade indicators. However, progress has been uneven across counties, with some regions experiencing stagnation or reversal in MTCT trends.

**Study context and rationale:** Kilifi County, located in the coastal region of Kenya, has an HIV prevalence of approximately 4.4% and is prioritized under the national eMTCT framework due to persistently elevated MTCT rates. Despite sustained PMTCT efforts, the county recorded an increase in MTCT from 7.7% in 2015 to 8.3% in 2018, with continued reports of HIV infections among HIV-exposed infants. Previous studies in Kenya and similar settings have largely focused on socio-demographic and clinical determinants of MTCT. However, fewer studies have systematically examined how failures in uptake and continuity of PMTCT services contribute to residual transmission, particularly using routinely collected program data. Understanding where and how mothers disengage from the PMTCT cascade is critical for designing targeted interventions to close service delivery gaps and advance progress toward elimination.

**Study objective:** this study aimed to identify risk factors associated with HIV infection among HIV-exposed infants by assessing uptake of key PMTCT services among HIV-positive mothers in Kilifi County. The study was guided by the hypothesis that HIV-exposed infants who acquire HIV are more likely to be born to mothers who defaulted at one or more critical steps of the PMTCT cascade.

**Specific objectives:** 1) to determine the association between antenatal care attendance among HIV-positive mothers and HIV infection status of their HIV-exposed infants; 2) to determine the association between maternal HIV testing during antenatal care and HIV infection status of HIV-exposed infants; 3) to determine the association between maternal antiretroviral therapy use, infant prophylaxis, and HIV infection status of HIV-exposed infants; 4) to determine the association between skilled facility-based delivery among HIV-positive mothers and HIV infection status of their HIV-exposed infants.

**Figure 1:**
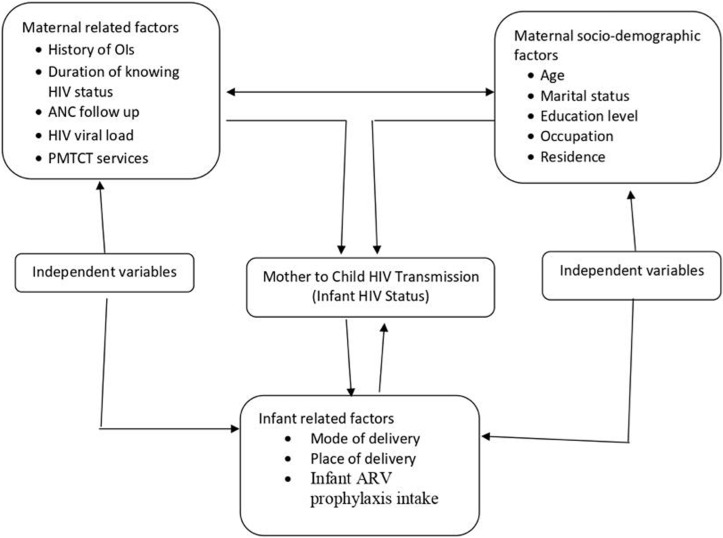
diagrammatic representation of the conceptual framework

**Conceptual framework:** the conceptual framework shows the interlinkage between the independent and dependent variables in this study. Based on the literature, there is an interconnection between maternal characteristics of HIV positive women and transmission of HIV to their children. Interventions focusing on the interlinkage of socio-demographic, the characteristics of mothers enrolled in the PMTCT program, and infant-related factors may prevent mother-to-child transmission of HIV, facilitating the elimination of mother-to-child HIV transmission (Figure 1).

## Methods

**Study design, setting, and period:** this study employed a retrospective unmatched case-control design using routinely collected health facility data. It was conducted in Kilifi County, located in the coastal region of Kenya. The county has an estimated population of approximately 1.5 million people and is administratively divided into seven sub-counties. At the time of the study, Kilifi County had 294 health facilities, of which 173 provided PMTCT services. Kilifi County is prioritized under Kenya´s elimination of mother-to-child transmission (eMTCT) framework due to persistently elevated rates of vertical HIV transmission. The study included HIV-positive mothers enrolled in PMTCT services and their HIV-exposed infants who had documented confirmatory HIV DNA PCR test results between July 2018 and June 2019. Data abstraction and analysis were conducted between December 2020 and September 2021.

**Study population:** the study population comprised HIV-positive mothers enrolled in PMTCT programs in Kilifi County and their index HIV-exposed infants. Mothers and infants were drawn from the same source population across public and private health facilities offering PMTCT services within the county.

**Case and control definition:** cases were defined as HIV-positive mothers enrolled in PMTCT services whose HIV-exposed infants were confirmed HIV-positive by HIV DNA PCR testing during the study period; controls were defined as HIV-positive mothers enrolled in PMTCT services whose HIV-exposed infants were confirmed HIV-negative by HIV DNA PCR testing during the same period.

**Sample size determination:** sample size was calculated using Epi Info version 3.01 (StatCalc) for an unmatched case-control study. The calculation assumed a 95% confidence level, 80% statistical power, and a case-to-control ratio of 1: 2. An anticipated odds ratio of 1.8 was used, based on findings from similar studies conducted in sub-Saharan Africa. The estimated proportion of exposure (≥4 PMTCT follow-up visits) was 32.4% among cases and 74.9% among controls. The minimum required sample size was 183 mother-infant pairs, comprising 61 cases and 122 controls.

**Sampling technique:** a stratified random sampling approach was employed. Health facilities providing PMTCT services were used as strata. The number of cases and controls selected from each facility was proportional to the facility´s PMTCT client load. Eligible case and control records were identified using electronic and paper-based medical registers. Simple random sampling was then used to select records within each stratum.

### Study variables

**Dependent variable:** infant HIV infection status, confirmed by HIV DNA PCR testing.

**Independent variables:** antenatal care attendance; maternal knowledge of HIV status prior to or during pregnancy; maternal antiretroviral therapy (ART) use during pregnancy and breastfeeding; infant antiretroviral prophylaxis; place of delivery. These variables represent key steps within the PMTCT cascade.

**Inclusion criteria:** HIV-positive women enrolled in PMTCT programs in Kilifi County; HIV-positive mothers whose infants had a documented confirmatory HIV DNA PCR test result (positive for cases, negative for controls) between July 2018 and June 2019.

**Data collection:** data was collected using a structured data abstraction tool designed to capture maternal socio-demographic characteristics, antenatal care attendance, delivery information, maternal ART history, infant prophylaxis, and infant HIV outcomes. Data were abstracted from antenatal care, maternity, postnatal, and PMTCT registers.

Data abstraction was conducted by trained clinical nurses with at least one year of experience in PMTCT service delivery. Extracted data were entered into Microsoft Excel, stored on password-protected computers, and securely backed up by the principal investigator. All personal identifiers, including names and clinic numbers, were excluded during data abstraction. Investigators did not have access to information that could identify individual participants.

**Statistical analysis:** data were cleaned and assessed for completeness using Microsoft Excel and analyzed using IBM SPSS Statistics version 21. Descriptive statistics were used to summarize categorical variables using frequencies and proportions. To address the study objectives, associations between PMTCT cascade indicators and infant HIV infection status were first assessed using chi-square tests. Variables with a p-value <0.2 at bivariate analysis were included in a multivariable binary logistic regression model to identify independent factors associated with vertical transmission of HIV. Results were reported as odds ratios (ORs) with 95% confidence intervals. Statistical significance was set at p < 0.05.

**Ethical considerations:** ethical approval was obtained from the Kenyatta National Hospital-University of Nairobi Ethics and Research Committee (KNH-UON ERC) (approval number: P374/07/2020). As this was a retrospective study using secondary data, a waiver of informed consent was granted. Permission to access facility records was obtained from the relevant county and facility authorities. Confidentiality and data security were maintained throughout the study.

## Results

**Characteristics of study participants:** a total of 161 HIV-exposed infants and their HIV-positive mothers enrolled in PMTCT services across selected health facilities in Kilifi County were included in the analysis. Of these infants, 59 were HIV-positive (cases), and 101 were HIV-negative (controls). Infant sex distribution was comparable between cases and controls, with 49% male and 51% female. Most infants (56%) were enrolled into the HIV Exposed Infant (HEI) program between 4 and 24 weeks of age. The distribution of age at enrolment was similar between HIV-positive and HIV-negative infants. Baseline characteristics of the infants are presented in Table 1.

**Table 1:** demographic characteristics of HIV exposed infants enrolled in the HIV exposed infant program in Kilifi County, Kenya, from July 2018 to June 2019, stratified by infant HIV test outcome (N=161)

Sex	Infant HIV test outcome					
	Positive (n=59)		Negative (n=101)		Total (n=160)	
Male	29	49%	50	50%	79	49%
Female	30	51%	51	50%	81	51%
**Age of infant at enrolment into HEI program (weeks)**	**Infant HIV test outcome**					
	**Positive (n=60)**		**Negative (n=101)**		**Total (n=161)**	
<4	10	17%	43	43%	53	33%
4-24	35	58%	55	55%	90	56%
25-36	8	13%	1	1%	9	6%
37-72	7	12%	0	0%	7	4%

HEI: HIV exposed infant

**Association between antenatal care attendance and infant HIV status:** overall, 88% of mothers attended at least one antenatal care (ANC) visit during pregnancy. All mothers whose infants were HIV-negative attended ANC, while one-third (33%) of mothers whose infants were HIV-positive did not attend any ANC visit. Maternal ANC attendance was significantly associated with infant HIV status (p<0.001), with non-attendance strongly associated with HIV infection among HIV-exposed infants. These findings are summarized in Table 2.

**Table 2:** maternal socio-demographic and clinical characteristics associated with HIV test outcomes among HIV exposed infants enrolled in the HIV exposed infant program in Kilifi County, Kenya, from July 2018 to June 2019 (N=161)

Mother attended ANC?	Infant HIV test outcome					
	Positive (n=57)		Negative (n=99)		Total (n=156)	
Yes	38	67%	99	100%	137	88%
No	19	33%	0	0%	19	12%
**HIV positive status is known**	**Positive (n=59)**		**Negative (n=101)**		**Total (n =160)**	
Yes	56	95%	96	95%	152	95%
No	3	5%	5	5%	8	5%
**Disclosed**	**Positive (n=59)**		**Negative (n=101)**		**Total (n=160)**	
Yes	45	76%	92	91%	137	86%
No	14	24%	9	9%	23	14%
**Received maternal HAART**	**Positive (n=60)**		**Negative (n=101)**		**Total (n=161)**	
HAART	23	38%	98	97%	121	75%
None	37	62%	3	3%	40	25%
**When HAART started**	**Positive (n=23)**		**Negative (n=98)**		**Total (n=121)**	
First trimester	5	23%	52	53%	57	47%
Second trimester	4	17%	17	17%	21	17%
Third trimester	3	13%	4	4%	7	6%
Not stated	11	47%	25	26%	36	30%
**Place of delivery**	**Positive (n=59)**		**Negative (n=100)**		**Total (n =159)**	
Home	25	42%	3	3%	28	18%
Hospital	34	58%	97	97%	131	82%
**Infant prophylaxis regimen**	**Positive (n=55)**		**Negative (n=101)**		**Total (n=161)**	
NVP	5	9%	13	13%	18	12%
NVP and AZT	50	91%	88	87%	138	88%

ANC: antenatal care; HAART: highly active antiretroviral therapy; NVP: nevirapine; AZT: zidovudine

**Association between maternal HIV testing during ANC and infant HIV status:** the majority of mothers (95%) were aware of their HIV status prior to or during ANC attendance, with no substantial difference between cases and controls. Maternal knowledge of HIV status alone was not significantly associated with infant HIV infection, indicating that awareness without timely engagement in PMTCT interventions was insufficient to prevent vertical transmission.

**Association between maternal ART use, infant prophylaxis, and infant HIV status:** overall, 75% of mothers received antiretroviral therapy (ART) during pregnancy. However, 62% of mothers whose infants were HIV-positive did not receive ART, compared to only 3% among mothers of HIV-negative infants. Receipt of maternal ART during ANC was strongly associated with favorable infant HIV outcomes (p < 0.001).

In logistic regression analysis, infants whose mothers received ART during pregnancy were significantly more likely to be HIV-negative (OR 52.55; 95% CI: 14.89-185.48). This association remained robust after adjustment for other PMTCT cascade factors (AOR 30.23; 95% CI: 7.56-120.96).

Most infants received antiretroviral prophylaxis, with 88% receiving both nevirapine and zidovudine. Infant prophylaxis coverage was high in both groups, with minimal differences between HIV-positive and HIV-negative infants. Infant-related variables are summarized in Table 3, while adjusted associations are presented in Table 4.

**Table 3:** infant-related clinical and programmatic characteristics of HIV exposed infants enrolled in the HIV exposed infant program in Kilifi County, Kenya, from July 2018 to June 2019 by infant HIV test outcome (N=161)

Entry point	Infant HIV test outcome					
	Positive (n=59)		Negative (n=98)		Total (n=157)	
Male	4	7%	3	3%	7	4%
Female	5	8%	1	1%	6	4%
**Age of infant at enrolment into HEI program (weeks)**	**Infant HIV test outcome**					
	**Positive (n=60)**		**Negative (n=98)**		**Total (n=158)**	
Initial 6 weeks/first contact	48	80%	94	96%	142	90%
Second PCR (6 months)	4	7%	3	3%	7	4%
Third PCR (12 months)	3	5%	0	0%	3	2%
Other time	5	8%	1	1%	6	4%
**Infant prophylaxis regimen**	**Infant HIV test outcome**					
	**Positive (n=55)**		**Negative (n=101)**		**Total (n=161)**	
NVP	5	9%	13	13%	18	12%
NVP and AZT	50	91%	88	87%	138	88%

HEI: HIV exposed infant; PCR: polymerase chain reaction; NVP: nevirapine; AZT: zidovudine

**Table 4:** bivariate and multivariate logistic regression analysis of factors associated with HIV infection among HIV-exposed infants enrolled in the HIV exposed infant program in Kilifi County, Kenya, from July 2018 to June 2019 (N=161)

Infant HIV test outcome							
Variable	Category	HIV positive (n, %)	HIV negative (n, %)	Total (n)	P-value	COR (95% CI)	AOR (95% CI)
Mother attended ANC?	Yes	3 (8.9%)	67 (91.1%)	70	-	-	-
	No	1 (5%)	19 (95%)	20	-	-	-
HIV positive status known?	Yes	5 (96%)	95 (95%)	152	0.970	1.03 (0.24-4.47)	0.74 (0.98-5.64)
	No	3 (5%)	5 (5%)	8	-	-	-
Disclosed HIV status?	Yes	45 (76%)	92 (91%)	137	0.010	3.18(1.28-7.90)*	6.27(1.20-20.05)**
	No	1 (24%)	9 (9%)	23	-	-	-
Received Maternal HAART in ANC	HAART	23 (38%)	98 (97%)	121	0.000	52.55(14.89-185.48)*	30.23(7.56-120.96)**
	None	37 (62%)	3 (3%)	40	-	-	-
Mode of delivery	SVD	58 (97%)	97 (96%)	155	0.839	0.84(0.15-4.71)*	0.53(0.03-9.65)**
	CS	2 (3%)	4 (4%)	6	-	-	-

ANC: antenatal care; HIV: human immunodeficiency virus; HAART: highly active antiretroviral therapy; SVD: spontaneous vaginal delivery; CS: caesarean section; COR: crude odd ratio; CI: confidence interval; AOR: adjusted odds ratio

**Association between maternal disclosure, skilled delivery, and infant HIV status:** disclosure of maternal HIV status to a partner was reported by 86% of mothers overall. Disclosure was more common among mothers of HIV-negative infants (91%) than among mothers of HIV-positive infants (76%). Infants whose mothers disclosed their HIV status were significantly more likely to be HIV-negative (OR 3.18; 95% CI: 1.28-7.90), and this association remained significant after adjustment (AOR 6.27; 95% CI: 1.20-20.05). Most mothers (82%) delivered in a health facility. However, home delivery was substantially more common among mothers of HIV-positive infants (42%) compared to those of HIV-negative infants (3%). Facility-based delivery was significantly associated with reduced risk of infant HIV infection.

## Discussion

This study demonstrates that persistent gaps along the PMTCT cascade remain the primary drivers of residual mother-to-child transmission of HIV in Kilifi County. Maternal ANC attendance, uptake of ART during pregnancy, disclosure of HIV status, and skilled facility-based delivery were key determinants of infant HIV outcomes.

The Kenya Demographic and Health Survey 2022 showed that 98% of women aged 15-49 years with a recent birth received at least one ANC visit from a skilled provider, with first ANC visit coverage remaining fairly stable around 83%-84% from 2019 to 2024 [6], with completion of four ANC visits showing persistent gaps at 61-62% retention.

Antenatal care (ANC) services represent the critical entry point for PMTCT interventions, including HIV testing, ART initiation, adherence counselling, and preparation for skilled delivery. In this study, ANC nonattendance was strongly associated with infant HIV infection, highlighting missed opportunities for early intervention. This finding is consistent with evidence from sub-Saharan Africa showing that ANC attendance substantially increases engagement in PMTCT services and reduces vertical transmission [2]. A study done in Brazil showed that lack of antenatal care and late diagnosis of HIV underlie substantial gaps in PMTCT Cascade [6]. No studies were found that contradicted this finding.

Although most mothers were aware of their HIV status prior to or during pregnancy, knowledge alone did not differentiate infant outcomes. This underscores the importance of translating HIV diagnosis into timely ART initiation and sustained viral suppression, rather than relying on awareness alone. This finding was similar to a study done in Western Kenya, which showed that 68% of women who had knowledge of their HIV-positive status enrolled in the PMTCT program were not on ART at the time of conception. For those on HAART, a significant proportion (14%) had unsuppressed viral load. This goes in line with our finding that awareness of HIV status alone may not contribute to prevention of vertical transmission [7]. This finding was contrary to a study done in South Africa that showed that women aware of their HIV positive status before the index pregnancy were more likely to enroll in the PMTCT program and test their infants early, thereby preventing transmission [8].

Maternal ART uptake emerged as the strongest protective factor against vertical transmission. Infants born to mothers who did not receive ART or initiated ART late were at markedly increased risk of HIV infection. Early initiation of ART, particularly during the first trimester, is well established to reduce maternal viral load and transmission risk, and the findings of this study reinforce this evidence within the local context of Kilifi County. Several studies have shown the importance of maternal HAART in the prevention of vertical transmission [9]. A study done in Nigeria showed that none of the exposed children whose mothers were on HAART became HIV positive [10]. A study done in Ethiopia showed that poor adherence to maternal HAART was associated with an increased risk of vertical transmission [11]. Disclosure of HIV status was independently associated with favorable infant outcomes, likely reflecting improved social support, adherence, and continuity of care among mothers who disclosed. This finding aligns with previous Kenyan studies linking disclosure to improved PMTCT engagement. It was established that women living with HIV who had not disclosed to anyone had the lowest levels of PMTCT utilization [12,13].

Labor and delivery pose the greatest transmission risk of HIV, with 10-20% of exposed infants being infected during this time. Our study demonstrated that facility-based delivery was associated with reduced transmission, while home delivery remained a significant risk factor. This finding is similar to other studies done, which established the importance of skilled delivery in the prevention of mother-to-child transmission [7,9,11]. Skilled delivery facilitates safer obstetric practices and timely infant prophylaxis, reducing intrapartum transmission risk.

**Study limitations:** this study relied on retrospective review of routine health facility records, which may have been affected by incomplete documentation or missing data. Additionally, information on maternal viral load and adherence measures was not consistently available, limiting assessment of their direct contribution to infant outcomes.

**Implications for practice:** strengthening early ANC attendance, ensuring immediate ART initiation for all pregnant women diagnosed with HIV, supporting disclosure, and promoting skilled facility-based delivery remain critical to achieving elimination of mother-to-child transmission in Kilifi County and similar high-burden settings.

## Conclusion

This study confirms that effective and continuous implementation of the PMTCT cascade substantially reduces mother-to-child transmission of HIV. Missed opportunities at critical steps, particularly antenatal care attendance, maternal ART initiation, disclosure of HIV status, and skilled delivery, significantly increased the risk of infant HIV infection. Addressing gaps in retention and continuity of care across the PMTCT cascade is essential for achieving elimination of vertical HIV transmission.

### What is known about this topic

- Mother-to-child transmission of HIV can be effectively prevented through comprehensive PMTCT interventions;
- Early maternal HIV diagnosis and timely ART initiation reduce the risk of vertical transmission;
- Sustained viral suppression during pregnancy and breastfeeding is essential for elimination of MTCT.

### What this study adds

- We found that failure to attend antenatal care, lack of maternal ART during pregnancy, non-disclosure of HIV status, and home delivery were independently associated with HIV infection among HIV-exposed infants in Kilifi County;
- We demonstrate that maternal ART uptake during pregnancy remains the strongest protective factor against vertical transmission in routine program settings;
- This study provides county-level evidence highlighting specific PMTCT cascade gaps that continue to drive residual mother-to-child transmission of HIV.
